# The use of non-prescribed antibiotics; prevalence estimates in low-and-middle-income countries. A systematic review and meta-analysis

**DOI:** 10.1186/s13690-020-00517-9

**Published:** 2021-01-03

**Authors:** Neusa F. Torres, Buyisile Chibi, Desmond Kuupiel, Vernon P. Solomon, Tivani P. Mashamba-Thompson, Lyn E. Middleton

**Affiliations:** 1 Instituto Superior de Ciências de Saúde (ISCISA), Maputo, Mozambique; 2 Discipline of Public Health Medicine, School of Nursing and Public Health, University of KwaZulu Natal, Durban, South Africa; 3 Discipline of Pharmaceutical Sciences, School of Health Sciences, University of KwaZulu Natal, Durban, South Africa; 4 Faculty of Health Sciences, University of Pretoria, Pretoria, South Africa

**Keywords:** Non-prescribed antibiotics, Prevalence, Self-medication, Sources, Meta-analysis, Antibiotic resistance, LMICs

## Abstract

**Background:**

The global increase in the utilization of non - prescribed antibiotics (NPA), is concerning, with high persistence within the low and middle-income countries (LMICs). With a negative impact on the health of individuals and communities the use of NPA paves the way to the  propagation of superbugs that potentially predisposes to changes in bacterial resistance patterns, antibiotic resistance (AR) and antimicrobial resistance (AMR). This study aimed at estimating through a systematic review and meta-analysis, the prevalence of NPA utilisation and describe its primary sources in LMICs.

**Methods:**

The study is a systematic review and meta-analysis which study protocol was registered in PROSPERO (CRD42017072954). The review used The Preferred Reporting Items for Systematic Review and Meta-Analysis (PRISMA) guidelines.  The studies searched in databases were deemed eligible if reported evidence of practices of self-medication with antibiotics (SMA) and the prevalence of NPA utilisation within adult participants from LMICs, published between 2007 to 2017. The pooled analyses were carried out using Meta XL statistical software. The pooled prevalence was calculated with a 95% confidence interval (CI). The risk of bias of the included studies was assessed using the Quality in Prognosis Studies (QUIPS) tool.

**Results:**

The review included a total of 11 cross-sectional studies, involving 5080 participants and conducted in LMICs from Asia (India, Laos, Nepal, Pakistan, Sri Lanka and Yemen), Latin America (Guatemala), Africa (Nigeria). All studies reported existing  practices of SMA, with reported prevalence ranging from 50% to 93,8%. The pooled prevalence of SMA was 78% (95% CI: 65–89%). The main sources of NPA were; pharmacies, family and friends, old prescriptions, home cabinet and leftover antibiotics.

**Conclusion:**

This study revealed a high prevalence of utilisation of NPA in the studied LMICs, these were found to be twice as high in women than men and those participants aged between 18 and 40 years old. The review suggests f considering broader qualitative and comprehensive contextuallized research to better understand the nuances of NPA use. These would be benefitial to uncover uncover gray areas, inform decisions, support the (re) design and implementation of multifaceted interventions towards antibiotic stewardship and conservancy in LMICs.

## Background

In the antibiotic post-era, antimicrobial resistance (AMR) and precisely antibiotic resistance (AR) has ceased to be a threat and has become a real public health problem. AMR and AR has been contributing significantly to the morbidity and mortality rates in many settings with emphasis to the low and middle-income countries (LMICs). Albeit the misuse or inappropriate utilization of antibiotics can cause hazardous, when correctly used, the crucial value of these drugs for the prevention and treatment of a variety of infections is undeniable. This value lead tantibiotics to be one the most frequently prescribed and used medicines worldwide [1–4]. While the World Health Organization (WHO) warns that two-thirds of antibiotics available in the pharmaceutical sector are used for self-medication [5], the fast-growing antibiotic utilisation mainly the misuse and overuse have been considered one of the chief contributors to a higher burden of AMR [1–3, 6]. This is also shown by the rates of antibiotic consumption that increased from approximately 50 billion to 70 billion units between the years 2000 and 2010 [4]. Antibiotic resistance occurs when bacteria evolve to protect themselves from antimicrobial agents; it occurs when bacteria changes in response to the utilization of antibiotics. Antibiotic resistance implies that the bacteria’s causing certain infections no longer respond to that specific antibiotic [7–9], leading the infectious ailment to continue untreated while, prolonging the morbidity status of the patient.

Furthermore, the unwise and frequent consumption of non-prescribed antibiotics (NPA) is concerning and reportedly persisting in LMICs [1, 10–17] with limited to non-existent robust mechanisms of health promotion and enforcement measures to control and limit the unnecessary utilisation of antibiotics [3, 18–22]. LMICs therefore, have been experiencing high and unfavorable trends in resistance [23]. Besides, most LMICs lack robust surveillance systems reports and data regarding the infection and AMR rates, as well as the proportion of antibiotics and antimicrobials used either by the health care workers or by the community. However, estimates from the WHO, consider the infection rates and AMR to be higher in LMICs than in other regions and is believed to be causing many more deaths [12, 24–26].

In this study, the use of NPA is described as the administration of antibiotics without health care professional oversight and prescription, for self-medication, the use of leftover from the previous course, the storage of antibiotics for emergency purposes and further use, the use of antibiotics recommended or advised by pharmacists or pharmacy personnel, sharing antibiotics or antibiotic prescriptions with friends or family members, all to treat self-perceived illnesses.

It has been documented that the accessibility, availability, and affordability of NPA, have been threatening health authorities containment strategies of AMR as recommended by WHO and other global health organizations [3, 12, 23, 27–29]. Moreover, behind the extensive consumption of antibiotics, numerous factors have been reported to drive inappropriate utilisation of antibiotics including cursory knowledge, limited and inconsistent knowledge about antibiotics, financial constraints, cultural beliefs on the curative power of antibiotics, prescribing and dispensing practices, financial constraints, conditions of health care facilities, health-seeking behavior and individual decision to be entitled of own health [10–12, 30–35].

The findings of our previously conducted systematic scoping review aimed at mapping evidence of the factors influencing the practices of self-medication with antibiotics (SMA) in LMICs emphasised a considerable high prevalence of SMA related with the level of education, monthly income, gender of participants, and also with the accessibility, affordability, and conditions of health care facilities [21]. Even though some studies have reported the prevalence of NPA in few different populations, evidence reporting the prevalence in most LMICs are scarce. A systematic review and metanalysis of 34 studies that intended to establish the burden, risk factors and effects of antimicrobial self-medication in LMICs between 2002 to 2012 reported an overall prevalence of antimicrobial self-medication of 38.8% (95% CI: 29.5–48.1). Although most of the included studies of this review assessed non-prescription use of antibacterial (17/34:50%) and antimalarials (5/34:14.7%) agents and not only antibiotics [36], this study has revealed a considerable high prevalence of NPA utilisation.

Given the above backdrop, this review aims at providing an assessment of the prevalence estimate for utilization of NPA and its sources in LMICs. This effort would assess, estimate the real burden of the problem in resource constaraint settings, providing a significant contribution in the generation of evidence-based information from resource constraint settings.

## Methods

In 2017 we developed and registered a review study protocol (CRD42017072954) in the International Prospective Register of Systematic Reviews (PROSPERO). We have also conducted a review on the factors influencing the practices of SMA, peer-reviewed and published [37] to which this metanalysis is a follow-up.

### Study design

We performed a systematic review and meta-analysis of published studies reporting the practices of SMA in order to assess the best available estimate of the prevalence of NPA utilisation in LMICs.

### Search strategy

The systematic review and meta-analysis were performed according to the PRISMA guidelines, and the guidance of Barendregt et al. The search was conducted from Web of Science; PubMed, Science Direct; Google Scholar, EBSCOhost and World Health Organization (WHO). The database search occurred from July 2017 to february 2018 using the following keywords: “self-medication”, “antibiotics”, “factors”, “prevalence”, “sources”, “non-prescribed” and “LMICs”. Boolean terms (AND, OR) were used to separate the keywords. Mesh (Medical Subject Headings) terms were also included. After removing duplicates, articles were assessed for inclusion into the review through title and abstract analysis. The titles of the articles returned were examined, and any that were irrelevant were excluded. Abstracts and the full text of the remaining articles were reviewed to find relevant studies that met the inclusion criteria. Additional relevant articles were identified by searching the reference lists of full-text articles. To retrieve studies that included adult participants, the authors used the term “adult” combined with the terms “self-medication” and “antibiotics”. Low-and-middle-income countries are defined in this study, according to the World Bank classification as those economies with a gross national income (GNI) per capita of 1046 or less to $4125 [38]. During the search period, the term LMICs was combined with the other search terms.

### Selection criteria

Inclusion and exclusion criteria were predefined to facilitate objective screening of articles. The studies were included if data on the prevalence of SMA were available. Adult population-based (cross-sectional) studies from LMICs published since from 2007 to 2017 reporting prevalence of SMA or that could be calculated from available data, were considered for inclusion. Reporting the prevalence of SMA did not necessarily have to be the primary outcome of the study. Systematic reviews, meta-analyses, conference presentations, and letters or correspondences were excluded.

### Selection of studies

The titles of the articles returned were independently examined for eligibility by two researchers (NFT, BC), and any that were irrelevant were excluded. Forward searching was done using the included references. Abstracts and the full text of the remaining articles were reviewed to find relevant studies that met the inclusion criteria. Studies were deemed eligible if they reported evidence of practices of SMA and or prevalence of NPA utilisation within adults from LMICs. We ensured that the second reviewer was blinded to the primary reviewer’s decisions, and checked the article selection, data extraction, and risk of bias assessment stages of this review. Any differences of opinion during the abstract screening were discussed until consensus was reached. During full article screening, a third reviewer (NG) was invited to resolve the discrepancies between the screening results of the primary and secondary reviewers.

### Assessment of risk of bias

Two review authors (NFT, BC) assessed the potential risk of bias in all of the included studies by using the Quality in Prognosis Studies (QUIPS) tool, adapted for cross-sectional studies. QUIPS is a Critical appraisal tool to assess and identify biases in prognostic studies. The tool help reviewers conducting systematic reviews and developing clinical practice guidelines and readers of such studies [39]. QUIPS tool has six essential domains that are considered when evaluating validity and bias in studies of prognostic factors namely; study participation, study attrition, prognostic factor measurement, confounding measurement and account, the outcome of measurement, and analysis and reporting [39]. The risk of bias of the included studies was assessed using two domains of the tool, namely; study participants and outcomes of measurement. This tool was chosen due to its relevance for included cross-sectional studies [39]. During the quality assessment, a third reviewer (NG) was invited to resolve the discrepancies between the primary and secondary reviewers. Appraisal of each domain provides a subjective assessment of the risk of bias (ranked as low, moderate, or high). Additionally, we conducted a simple assessment of publication and general bias.

### Data extraction

The full-text review was performed for all the selected articles and data extracted and sorted into the following variables: name of the study, leading author, data on prevalence as well as a percentage of the number of participants indulging in SMA were recorded. The data were extracted independently by two reviewers (NFT, BC), who extracted relevant information related to the research questions using a standardized data extraction sheet. The data extracted from all eligible studies included authors’ and date, study objectives, sample size, the prevalence of SMA, sources of NPA and the main findings. A data extraction form was also used to captured data relevant to the assessment of the risk of bias.

### Data synthesis

We initially conducted a descriptive analysis of the studies. Heterogeneity between estimates was assessed using the I^2^ statistic, which describes the percentage of variation, not because of sampling error across the included studies. We interpreted the I^2^ value above 75% as high heterogeneity. The Meta XL was employed to conduct a meta-analysis using a random-effects model to account for heterogeneity. A pooled prevalence figure was calculated with a 95% confidence interval (CI). Barendregt et al., (2013) stated that when the estimate for a study tends towards either 0% or 100%, the variance for that study moves towards zero and its weight will be overestimated [40]. However, we conducted the meta-analysis with prevalence estimates that had been transformed using the double arcsine method [40]. The final pooled result and 95% CIs were back transformed to simplify results interpretation. Where studies allowed, we descriptively compared prevalence estimates by age, gender and study settings of the included studies. We also assessed the data collection method by comparing studies, if data were collected by employing a self-completed questionnaire or if collected using a method that requires the assistance of the researcher (interview or telephone questionnaire). The primary outcome of this review is the prevalence of NPA utilisation, however, considering its importance, the main sources of NPA were included in a qualitatively synthesised form as a secondary outcome due to the non-feasibility of the meta-analysis and odds ratios.

## Results

### Screening results

**Fig. 1:**
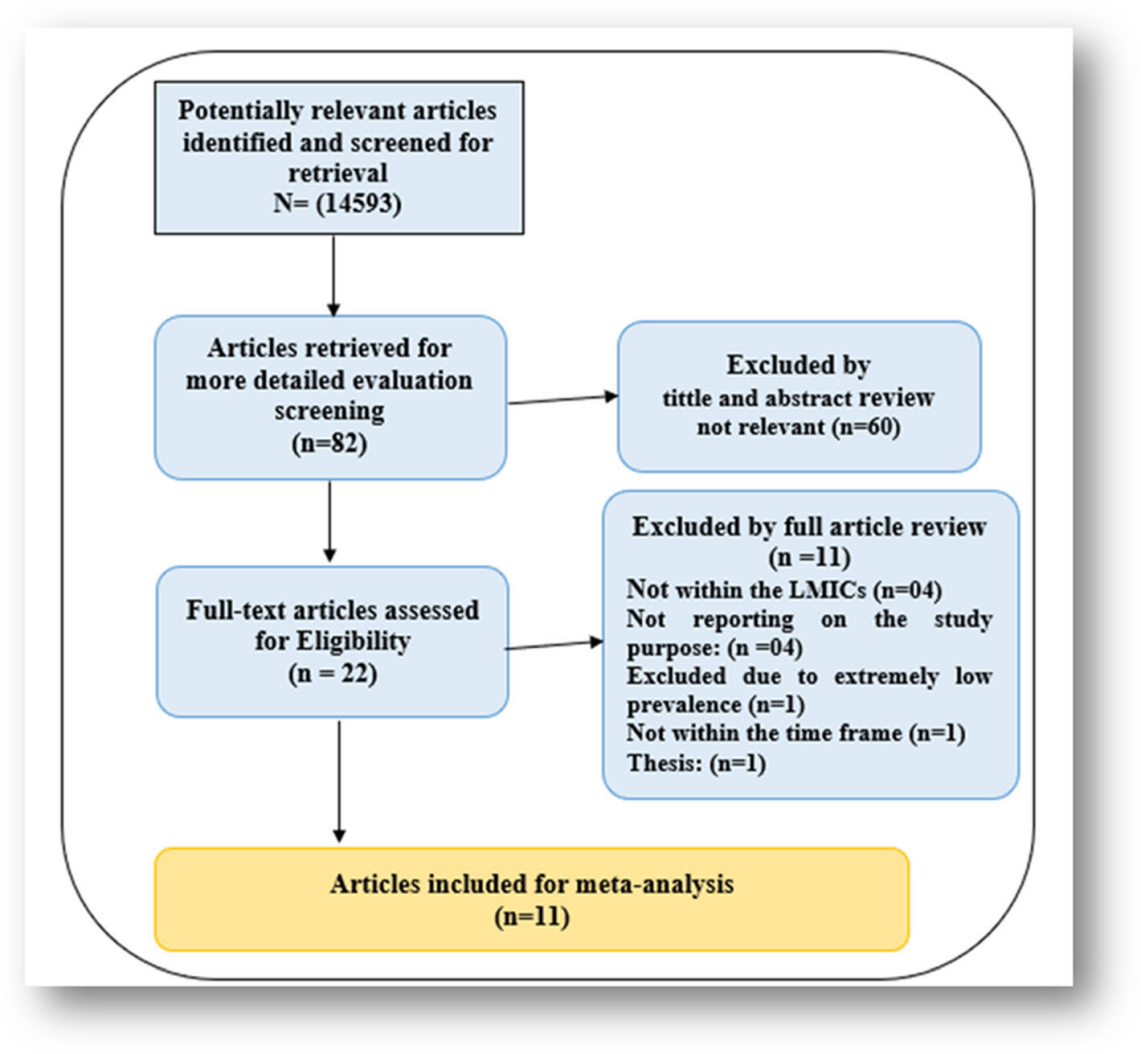
Study Selection

The search yielded a total of 14,593 potentially relevant titles. After undertaking exclusions based on selection criteria, removing duplicates, letters, thesis, dissertations and commentaries, these were reduced to 82 eligible then exported to the EndNote Library, where abstract screening was undertaken. A total of 60 studies were excluded at abstract screening, as they did not meet the study’s inclusion criteria. Therefore, 22 studies were included for full article screening, and eleven were excluded due to non-reporting prevalence of SMA or any data that could allow calculation. In the final, 11 studies (prevalence estimates) were included for data extraction. Some of the excluded studies at title, abstract and full article screening were used in our background and discussion sections. The results of the study selection are presented in Fig. 1.

### Characteristics of included studies

All included studies were conducted in nine LMICs countries and published between 2007 and 2017. Included studies were all descriptive cross-sectional in terms of study design [30–34, 41–46]. Seven of the included studies were conducted in India, Laos, Nepal, Pakistan, Sri Lanka and Yemen [30, 32, 34, 41, 44–46], two were conducted in Nigeria [33, 42] and another two in Guatemala [31, 43]. In terms of the study settings, five of the included studies were conducted in urban settings [34, 42–44, 46], while three were carried out in rural settings [30, 33, 45] and another three conducted in both rural and urban settings [32, 41, 43]. The study settings included universities [34, 44], a hospital [30], a primary health care center [33], pharmacies [31, 41, 43] and households [32, 45]. The sample size of all included studies ranged from 150 to 1827 participants [30–34, 41–46]. Participant’s age ranged from 18 to 69 years old with female participants predominant in ten of the studies [30–32, 34, 42–47].

Few included studies reported prevalence according to sex, age and setting of the participants. In all included studies, the minimum age for the study population was 18 years and the maximum 80 years old. Two studies presented age banded data, and these demonstrated that SMA prevalence occurs around age 18 to 40 years and over, continually increasing [33, 41].

Two articles presented prevalence figures by gender of  the participants. The prevalence was higher for women in all studies; except for one study, where male participants dominated the prevalence of NPA with 90% against 83% of the female counterpart [41]. Sex variation was also reported in one study conducted in a rural setting (*p* = 0.04; 64.9 vs 35.1), where it was evident that there was a significant difference between self-medication practiced by males with the one practiced by females [30]. One study made comparisons between populations from urban and rural settings, where it was observed appreciable differences between the two settings with a higher prevalence within participants from the rural setting, 79 and 77% for the urban setting [31]. Another study reported variations between participants from the lower and higher socio-economic areas of the city. Significant differences in the prevalence of 93% for the lower socio-economic setting and 73% for the higher economic setting were reported [43]. The summary characteristics of included studies are presented in Table 1.

**Table 1:** **Summary of included studies**

Author/Date	Country	Objective	Sample Size	Prevalence	CI	Sources NPA	Main findings
**Abdulraheem, et al., 2016** [33]	Nigeria - Rural	To estimate the prevalence and identify factors associated with to SMA	150	63%	95%	Drug stores Pharmacy Friends/family Remnant stock	Easy access to information from drug indices, medical literature and colleagues gives the sense of control. Misplaced lead to inappropriate self-medication.
**Aditya, et al., 2013** [34]	India – Urban	To compare features of SMA among undergraduate dental students.	1150	82.2%	95%	Pharmacy Home cabinet	Level of education significantly influenced. Males more prone to self-medication than females. Economic factors – SMA is cheaper and affordable.
**Albawani, et al., 2016** [41]	Sana City - Yemen Rural/urban	To determine the prevalence of SMA and its associated risk factors.	363	87.1%	95%	Community drug dispensers Friends/family Remnant stock	Poor medication knowledge lack of awareness, Poor dispensing control
**Bilal, et al.,2016** [30]	Sindh City - Paquistan Rural	To evaluate the prevalence and practice of self-medication with antibiotics	400	81.25%	95%	Pharmacies Corner stores Home cabinet	Population, self-medicating had low level of education, with almost half of them uneducated, and mostly belonging to the low socioeconomic class.
**Ramay, et al., 2015** [48]	Guatemala-Rural/Urban	To compare the magnitude of SMA and the characteristics of Those who self-medicate with antibiotics in four pharmacies	418	78%	95%	Pharmacy left over from previous prescriptions Family member Friend Publicity, Internet	Differences in socio-economic characteristics have been cited as a determinant for self-medication practices Motives for SMA were centered on the cost of medical visits.
**Ramay, et al., 2017** [31]	Guatemala City Urban	To understand the practice of SMA in four Guatemalan private pharmacies by comparing the characteristics of SMA in Guatemala, sources of information used, perceived effects of SMA and motives	230	79%	80%	Pharmacies Supermarket Corner stores From home (previously purchased) Pharmacy employee Family, Friend	High proportions of self-medication were similar in both pharmacies, despite the differences in monthly income and educational level.
**Israel, et al., 2015** [42, 49]	Nigeria Urban	To estimate the prevalence of SMA and evaluating the socio-demographic factors associated with the practice	471	93.9%	NS	family and friend’s leftovers Community Pharmacy Hospital pharmacy	Respondents with higher educational level showed higher prevalence of SMA than those with lower educational qualification. SMA because of lack of funds to purchase drugs or pay hospital bills. Familiarity of the population to Beta lactam class this class of antibiotics could contribute to their misuse and abuse. The high consultation of patent medicine dealers for drug and health information is appalling and should be discouraged.
**Senadheera, G. et al., 2017** [45]	Sri lanka - Rural	To determine the period prevalence of SMA in the Colombo District and to describe the reasons for SMA, its utilization pattern and socio-economical determinants	431	80.4%.	95%	Pharmacy Left over previous prescription Given to another person Left over from previous prescription Contact in hospital Friend	The study has provided data about the practice of SMA in Colombo district and identified an important area to be addressed in antibiotic stewardship programmes.
**Sah, A. et al. 2016** [50]	Nepal- Urban	To estimate the prevalence of SMA among nursing students and evaluate factors associated.	500	91%	95%	Previous experience of same illness Seen previously doctor prescription Advice from colleagues and seniors	It was reported using antibiotics for an inappropriate duration of time, and few of them knew the dosage of drugs used.
**Shah, S. et al., 2014** [46]	Pakistan – Urban	To provide the prevalence of self-medication with antibiotics amongst the university students of Karachi.	431	80.4%	NS	NS	The prevalence of self-medication with antibiotics among the non-medical university students was high despite the awareness of adverse effects. Antibiotic resistance was a relatively unknown terminology.
**Shiavong, A. et al., 2017** [51]	Laos- Urban/Rural	To describe antimicrobial self-medication for reproductive tract infections (RTI) including sexually transmitted infections (STI), and to explore the understanding and use of health information among the adult population	500	91%	NS	Pharmacies, Following previous treatment Nurse Drug seller Friend Parents/relative	More than three quarters of respondents, self-medicating for RTI/STI with antimicrobials, used inappropriate drugs bought from private pharmacies. There is a need to improve RTI/STI management, including health promotion, through interventions at community level, and to health providers, including private drug sellers.

*NS* Not specified

Risk of bias assessment

### Risk of bias

The Quality in Prognosis Studies (QUIPS) tool was adapted for cross-sectional studies. The risk of bias of the included studies was assessed using two domains of the tool, namely; study participants and outcomes of measurement, due to the relevance of the tool for included cross-sectional studies [39]. Appraisal of each domain provides a subjective assessment of the risk of bias (ranked as low, moderate, or high).

#### *Study participants bias*

In this way, 63.6% (*n* = 7) studies were at low risk of bias for study participation [30–33, 41–43]. In these studies, the sample was randomly selected, the period of recruitment and place of recruitment were also included, which demonstrated that the sample was representative of the study population. On the other hand, 27.2% (*n* = 3) studies were considered at high risk of participation bias [34, 44, 45], due to the recruitment from a non-representative sampling frame, in one of the studies, the selection of the sample was not clear, so whom to approach was still at the discretion of the interviewer and was therefore likely to be biased [44]. Finally, 18.1% (*n* = 2) studies had a moderate risk due to the small sample size, despite the high response rate [32, 46].

#### *The outcome of measurement bias*

At high risk of the outcome of measurement bias due to non-prevalence estimates specified were 18, 1% (n = 2) studies [34, 44], in these studies the data collected were self-reported, which may introduce bias in the behaviors of the respondents. One study had moderate risk since the prevalence of SMA was calculated by dividing the number of respondents who used an antibiotic without a valid prescription (SMA) in the last three months by the total of respondents [52]. Additionally, 72,7% (*n* = 8) studies were at low risk, where the prevalence SMA estimates were calculated from the data extrapolated from a subsample, there was a clear use of a study-specific questionnaire [30–33, 41–43, 46]. One study pre-tested structured interview form containing both closed and open-ended questions [32], also in one study the participants were given the option to answering the questionnaire themselves or having the researcher filling it based on the respondents’ verbal responses [30]. A summary data of the risk of bias for each included articles are provided in Tables 2, 3, 4, and 5.

**Table 2:** Guidelines for assessing risk of bias – based on study participation and outcome measurement domains of the QUIPS tool [39]

Potential bias	Items to be considered for assessment of potential bias
**Study participation**	
Does the study sample represent the population of interest on key characteristics sufficient to limit potential bias to the results?	**Target population:** The source population or population of interest is adequately described for key characteristics.
	**Sampling:** The sampling frame and recruitment are adequately described, possibly including methods to identify the sample (number and type used, e.g. referral patterns in health care), period of recruitment, and place of recruitment (setting and geographic location). The sampling frame and procedures used to sample subjects should not lead to selection of participants that are systematiclly different from eligible non-participants.
	**Inclusion criteria:** Inclusion and exclusion criteria are adequately described (e.g. including explicit diagnostic criteria or “zero time” description). Inclusion/exclusion criteria should not select participants that are systematically different from eligible non-participants.
	**Baseline study population:** The baseline study sample (i.e. individuals entering the study) is adequately described for key characteristics.
	**Adequate study participation:** There is adequate participation in the study by eligible individuals. Studies should report factors associated with non-response, quantify and interpret these associations to determine if it is a selective sample. For example, a low participation raises suspicion that there may be a barrier to participating that may influence outcomes.
**Outcome measurement**	
Is the outcome of interest adequately measured in study participants sufficeint to limit potential bias.	**Definition of outcome:** A clear definition of the outcome of interest is provided, including duration of follow-up and level and extent of the outcome construct.
	**Valid and reliable measure of outcome:** The outcome measure and method used are adequately valid and reliable to limit misclassification bias (e.g., may include relevant outside sources of information on measurement properties, also characteristics, such as blind measurement and confirmation of outcome with valid and reliable test). Whenever possible, validated instruments should be used.
	**Method and setting of outcome measurement:** The method and setting of measurement are the same for all study participants. The measurement approach, timing, and setting of assessment should be standardised across subjects, or conducted in a way that limits systematically different measurement. If there are differences, this should be reported and the implications should be considered.
	**Estimation of population parameters:** Estimates of population parameters should be calculated using data observed in the whole sample, not extrapolated from rates observed in a sub-sample.

**Table 3:** Risk of study participation and outcome measurement bias - the QUIPS tool

1	**Abdulraheem et al., 2016** [32, 48]	**Low:** Despite the study being conducted in a Health facility the sample size and the response rate are very high with 1150 and 95,5% respectfully.	**Low:** Used a descriptive and comparative statistical data analysis was processed with the SPSS. Simple and multiple logistic regression models were used to evaluate associations between participant characteristics and reported usage of antibiotics.
2	**Aditya, S. et al. 2013** [34]	**High:** although high participation rate of 94.8 and 97.4%, clear comparison of responders vs. non-responders. The sample was not representative of the study population. We also question the self-administered questionnaire	**High:** Measurement of outcome is valid, reliable and similar for all subjects. However, data collected were self-reported which may introduce some bias in the behaviors of the respondents studied.
3	**Albawani, S.et al., 2016** [41]	**Low:** A convenience sampling method was used to distribute 363 questionnaires were to consumers attending 10 selected community pharmacies. The pharmacies were carefully selected to represent different areas in Sana’a City.	**Low:** Clear operationalization of outcome measure, the bivariate analysis was used to identify the risk factors associated with the use of antibiotics during self-medication Access.
4	**Bilal, M. et al.,2016** [30]	**Low:** Although the sample was selected from hospital patients, the questionnaire went through a pilot phase in which 30 people who conformed to the inclusion criteria. Thesampling was from an appropriate sampling frame.	**Low:** Measurement of outcome is similar for all subjects. The questionnaire was then given to the volunteers to fill out. It contained 5 sections (A, B, C, D and E). Participants were given the option of answering the questionnaire themselves or having the researcher fill it based on verbal responses.was validated
5	**Israel, E. et al., 2015** [42, 49]	**Low:** The sample size was calculated according to a formula described by Badger et al. The questionnaires were randomly distributed to 526 civil servants based on the various ministries, departments, and units. The response rate was 89.5%.	**Low:** the outcome of measurement was conducted in a similar way, additionally the results were analysed by one-way ANOVA, using SPSS, all data were expressed as mean and difference between groups were considered significant at *P* = .05.
6	**Ramay, B. et al., 2015** [48]	**Low:** Good participation rate, good participation groups. The Sample size was calculated for each pharmacy using Epidat 4.0 based on a population of 350 patients arriving to the pharmacy weekly, assuming that 50% of the population self-medicates, a precision of 5% and a 95% confidence level.	**Low:** Data was collected by a questionnaire that was designed based on instruments used in previous studies. The questionnaire was validated by interviewing 20 customers with .
7	**Ramay, B. et al., 2016** [31]	**Low:** Only customers arriving to pharmacies to purchase antibiotics without a prescription were invited to participate in the study, and the inclusion criteria as well as the age of the participants was considered. There is a reasonable, response rate of 60%.	**Low:** Measure of outcome similar for all subjects, criteria are clearly stated.
8	**Sah et al., 2016** [50]	**High:** Although the response rate was 99% the selection of the nursing students was not clear, so who to approach was still at the discretion of the interviewer and was therefore likely to be biased.	**High:** Measure of outcome was similar for all subjects. Although comprised of two components. The questionnaire was too short considering the objectives of the study.
9	**Senadheera, et al, 2017** [46]	**High:** Sample size was determined by the formula to estimate a population proportion and the prevalence of SMA was assumed to be 50%.	**Moderate:** Prevalence of SMA was calculated by dividing the number who used an antibiotic without a valid prescription (SMA) in the last 3 months by the total respondent. The estimated prevalence to SMA was calculated within 5% with 95% confidence. This might not a reflect of the answers of the participants.
10	**Shah, S. et al., 2014**	**Moderate:** The sample was representative it was calculate based on previous studies that had reported the SMA prevalence’s. However, the study included all male and female students enrolled in undergraduate or postgraduate programs in universities of Karachi who understood English. This might incur a certain level of bias.	**Moderate:** Clear use of a study specific questionnaire that used closed questions.
11	**Shiavong, A. et al., 2017** [51]	**Moderate:** Using ordinary sample size calculation the authors (estimated prevalence 50%, precision 5%, confidence interval 95%), additionally 10 villages were randomly selected for the household survey.	**Low:** A pretested structured interview form was performed, and the final tool contained both closed and open-ended questions was considered.

**Table 4:** Summary of Risk of bias assessment

Author/Date	Risk of study participation bias	Risk of Outcome of Measurement bias
**Abdulraheem, I. et al., 2016** [32, 48]	Low	Low
**Aditya, S. et al. 2013** [34]	High	High
**Albawani, S.et al., 2016** [41]	Low	Low
**Bilal, M. et al.,2016** [30]	Low	Low
**Ramay, B. et al., 2015** [48]	Low	Low
**Ramay, B. et al., 2016** [31]	Low	Low
**Israel, E. et al., 2015** [42, 49]	Low	Low
**Senadheera, G. et al., 2017**	High	Moderate
**Sah, A. et al. 2016** [50]	High	High
**Shah, S. et al., 2014** [46]	Moderate	Moderate
**Shiavong, A. et al., 2017** [51]	Low	Low

**Table 5:** Publication Bias – Journal Impact factors

Author/Date	Journal Name	Impact factor	Risk of bias
**Abdulraheem, I. et al., 2016** [32, 48]	British Journal of Pharmaceutical Research	Unknown	High
**Aditya, S. et al. 2013** [34]	International Journal of Pharmaceutical Sciences and Research	1.23	Low
**Albawani, S.et al., 2016** [41]	Value in Health	5.03	Low
**Bilal, M. et al.,2016** [30]	Journal of clinical and diagnostic research: JCDR	1.23	Low
**Ramay, B. et al., 2015** [48]	BMC Pharmacology and Toxicology	1.77	Low
**Ramay, B. et al., 2016** [31]	Instituto de Investigaciones Químicas y Biológicas · Facultad de Ciências Químicas y Farmacia Universidad de San Carlos de Guatemala- Cientific Journal	Unknown	High
**Israel, E. et al., 2015** [42, 49]	Journal of Advances in Medical and Pharmaceutical Sciences	Unknown	High
**Senadheera, G. et al., 2017**	Ceylon Medical Journal	0.36	Moderate
**Sah, A. et al. 2016** [50]	International Journal of Pharma Sciences and Research (IJPSR)	0,38	Moderate
**Shah, S. et al., 2014** [46]	BMC Pharmacology and Toxicology	1.771	Low
**Shiavong, A. et al., 2017** [51]	Sex Transm Infect	3.346	Low

#### *Publication bias*

Within the included articles, eight out of eleven were published in international journals with impact factors ranging from 0.36 to 5.04 [30–32, 34, 41, 44, 46, 52]. One article was published in a local university journal [43] and in three articles the impact factor for the journals was unknown [33, 42, 43]. The risk of bias in all studies was considered sufficiently low to be included in this metanalysis based on the impact factors of the majority of the journals and the citation score at google scholar. General bias (reporting on ethical approval, conflict of interest) was considered low in all eleven studies, with all studies seeking and acquiring local ethical approval and authors declaring not having a conflict of interests.

#### *Degree of agreement*

Following full article screening, there was 63.64% agreement versus 70.25% expected by chance which constitutes a poor agreement between screeners (Kappa statistic = − 0.2 and *p*-value > 0.05). We have invited a third screener to resolve the discrepancies between the initials screeners’ results.

### Meta-analysis

#### The estimated prevalence of NPA utilisation

**Fig. 2:**
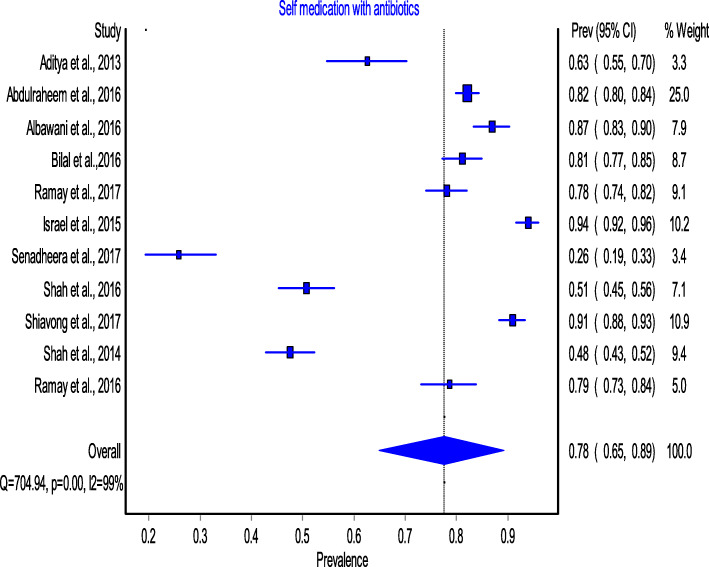
Pooled Prevalence of non-prescribed antibiotic utilization

Eleven cross-sectional studies reporting NPA antibiotic usage (*n* = 4498) and antibiotic self-medication (*n* = 3498) were included in this meta-analysis. The prevalence of NPA utilisation was reported by all included studies [30–34, 41–46]. The prevalence reported by each study ranged from 50% to 93,8%. The results show that the pooled prevalence of NPA utilisation in LMICS was 78% (95% CI: 65–89%). While Table 1. shows the prevalence reported by each study, Fig. 2. shows the pooled prevalence after performing the meta-analysis.

#### The main sources of NPA

The accessibility and availability of antibiotics for self-medication was evaluated in the included studies as one of the predisposing conditions influencing NPA utilisation among the participants. Within the included studies, information and advice on the utilisation of NPA were obtained from various sources including family members and friends [31, 33, 41–43, 53], city or community pharmacies [30, 31, 33, 34, 41–43, 45, 53], old prescriptions re-utilised at the pharmacies [41, 43–45], leftovers from previous prescriptions or sickness events [31, 42, 45], drug dispensers and drug stores [30, 42, 53], home cabinet [30, 33, 34], supplied by health care workers [44, 45, 53] and following advertisements, magazines and internet [31].

## Discussion

### Prevalence of NPA

The purpose of this study was to provide an assessment of the prevalence estimate of the utilisation of NPA and its sources in LMICs, according to the PRISMA statement. While significant efforts are putting in place to globally control the utilisation of antibiotics at all levels, this review, estimates the prevalence of NPA utilisation in LMICs as 78.8%. This prevalence differs substantially with the prevalence estimate of 38,8% reported in a previous review on global antimicrobial self-medication in LMICs published in 2015 [36]. We have noticed a large difference between the two reviews, however, the type of the reviews, the search terms, the time interval of the included studies, and the review questions may have influenced in the prevalence’s of SMA reported by the two reviews. For example, the first review included studies conducted between 2002 and 2012 and included not only antibiotics but also antimicrobial, antibacterial and antimalarial drugs. Moreover, due to the meta-analyses methodology, for some studies, our review estimated the prevalence based on the proportion of study participants. These aspects might have influenced the very large differences between the two reviews.

The age of participants determined the prevalence of NPA utilisation in the studied LMICs. The same was to gender and study setting, with women and those participants aged 18 to 40 years old having higher rates. Owour et al., (2015) reported similar results [54] in a study of SMA in Kenya. This is concerning since it is the women of the household, rather than the men, who could play an essential role in preventing SMA, especially in children and young people. Considering the active role of women within the family’s health, nutrition and care. The findings revealed that participants aged 18 to 40 years old were more prone to utilise NPA. This concurs with Afolabi, 2008 and Ekambi et al., (2019), whose findings observe that while the prevalence of NPA is distributed among the younger population, it is significantly higher among patients aged 41 years and older [55].

This study noticed some prevalence differences between the socio-economic groups of the study participants. The findings show high prevalence among low socio-economic class participants (with less level of knowledge and less livelihood) comparing to the high socio-economic class group. These findings are corroborated by Kurniawan et al., (2017), who stated that the utilisation of NPA was more prevalent in families with less income [56]. It is also essential to consider that respondents with more deficient knowledge have a higher probability of practicing SMA. Therefore, to prevent irrational and NPA utilisation, contextual and multifaceted educational programs aimed to improve public knowledge and promote responsible self-medication are needed.

### The sources of NPA

The review shows that pharmacies were the primary sources of NPA. These findings are similar to the results from studies carried out in Turkey [57] and the United Arab Emirates [58]. Moreover, according to Puspitasari et al., (2011), people may prefer to purchase non prescribed antibiotics in pharmacies because of quality assurance of the antibiotics and advice provided by pharmacy personnel [59]. Moreover, in a Jordanian study, about 53% of the NPA were dispensed by community pharmacists [60], whereas a study by Eticha et al., (2014), found it as high as 83% of NPA being dispensed in Ethiopia [18]. The availability and accessibility of antibiotics at pharmacies prompt clients to engage in the and inappropriate use of antibiotics. Considering the importance of reliable information on how to take the antibiotics, the adverse effects, the drug interaction and contraindications, this has the potential to expose the patients to a variety of health risk.

Family members and friends play an important role in influencing other’s behaviour and pratices. The included studies, reported pratices of SMA influenced by family members, neighbours and friends such as sharing prescriptions and leftovers antibiotics, advising antibiotics to treat certain health problems, administering NPA to a child, using leftovers antibiotics for next sickness events and home storage of antibiotics for future utilisation. The above-mentioned practices represent a drawback for the health care system as inappropriate antibiotic use paves the way to the development of AMR and AR which not only increases the number of bedridden sick people, the costs for purchasing more effective antibiotics, the costs for treatment of resistant infections but also increases the mortality rates [5, 61]. Therefore, making use and taking advantage of the influencing role of family members and friends to disseminate appropriate knowledge and better utilization of medicines and antibiotics in particular, is paramount.

According to antibiotic dispensing guidelines in the majority of the LMICs, antibiotics should be dispensed against a valid prescription. Additionally, each prescription should be used for the related treatment of the specific health condition as the physician prescribed it. Nevertheless, this review found a significant utilisation of old prescriptions of antibiotics for new sickness events as one of the sources of NPA stimulating the unwise use.

Due to their privileged position as patient’s attendants, medicine dispenser and adviser, pharmacists possess adequate biomedical knowledge on antibiotic indication and use. Provided with adequate knowledge and skills, pharmacists could play an essential role in educating patients, families, and communities, by making efforts to rationalise antibiotic utilization by preventing antibiotic sales without prescriptions. They could be the health promoters in nature, educating, informing each client on the risks of the utilisation of NPA and influence their behaviour by strictly dispense prescribed antibiotics. Therefore, refreshing training for pharmacist and other health care workers simultaneously with better supervision of prescribing and dispensing practices are crucial action to minimise the use of NPA in LMICs and contain the AMR and AR. Yet, robust regulatory enforcement and supervision combined by community awareness campaigns are required to promote the excellent sale and use of antibiotics. It is essential to be alert by promoting health literacy among communities and monitor the relevant factors that influence SMA towards antibiotic stewardship and conservancy.

#### Strengths and limitations

To our knowledge, this is the first meta-analysis systematically reviewing studies reporting the prevalence of utilisation of NPA and self-medication practices conducted in LMICs in the last ten years. Strengths of the current study are the systematic approach in which we have included descriptive cross-sectional studies. This study points out that evidence related to the utilisation of NPA is unequivocal. Additionally, by qualitatively synthesising the secondary outcome, the review evidences the primary sources of acquiring NPA reported.. Another strength of this study is the systematic and exhaustive search for relevant studies which helped in the identification of a considerate number of studies.. The manual identification of references in the texts of articles allowed additional articles to be found. The study followed transparent screening processes using keywords, which were guided by study Population Exposure and Outcome (PEO) nomenclature. A thorough data search using Boolean and MeSH terms was conducted during the literature search to increase the chances of finding eligible studies for inclusion in this review.

Despite the above-mentioned strengths, this review has limitations which should not be overlooked. First, summarising the prevalence of self-medication with antibiotics was not the main objective of most of the primary studies included. This might influence the accuracy of reporting these practices. Secondly, there was a potential for bias in the studies included due to the method of analysis, recall period, selection and social desirability. This influences the findings of the primary studies. For example, the frequency of usage of NPA was mainly assessed in most of the studies using self-reported and self-administered questionnaires, a method that runs the risk of recall bias and obtaining socially desirable responses. Nonetheless, with such an extensive review of a topic, it seems reasonable to conclude that the included studies present a sensible reflection of the general population’s prevalence of NPA utilisation in LMICs.

#### Implications for practice

With studies evidencing that the practice of  SMA has been driven by a variety of factors [12, 16, 21, 22, 27, 29, 62], the utilization of NPA seems to be part of the routine of management of self-diagnosed health problems. In the context of the current global problem of AMR, this review highlights the need to comprehend the complexity and the dynamic of the utilisation of antibiotics at the community level. This can be tackled by conducting research that focuses on community knowledge and practices regarding antibiotics, health-seeking behavior related to the practice of SMA and pharmacist’s dispensing practices.

Empowering pharmacy clients, patients and the general public, especially women with appropriated knowledge regarding the use of antibiotics, is needed. As evidence shows high participation of women in the utilisation of NPA, here is another opportunity for public health programs to target women and provide them with the appropriated information. Moreover, evidence shows that the accessibility and availability of antibiotics, the economic interests of pharmacies and the role of pharmacists added to the health-seeking behavior of individuals and communities, continuously poses a challenge for better control the use of antibiotics. All key stakeholders ought to engage and commit towards addressing the problem of NPA utilisation by (re) designing health promotion and education strategies to ensure user-centred outcomes, sustainability, and contextualization of the programs in LMICs.

#### Future research

Future research would be more beneficial if focusing on the prescribing and dispensing practices and the role of pharmacists and drug sellers, to assess the challenges faced by the pharmacists and inform more specific actions. It is therefore paramount to consider the influence of social, cultural and cognitively rooted influences on health-risk behaviour. Qualitative, descriptive and comprehensive studies that aim at understanding and describing the nuances of NPA utilisation are required to adequately assess the barriers and facilitators to the appropriate use of antibiotics within the LMICS. Impact studies both in urban and community settings aimed at contextually measuring the magnitude of the problem within the health care system need to be considered. Also impact studies of the existing programs would be beneficial to map the gaps, conduct research that generates evidence to inform decisions, guide the development and implementation of more effective health promotion strategies towards antibiotic stewardship and conservancy in LMICs..

## Conclusion

This systematic review and meta-analysis study summarized the evidence of the pratices of SMA, by providing prevalence estimates of the utilisation of NPA, these being twice as high in women than men. High prevalence was also reported in those participants aged between 18 and 40 years old. Notwithstanding the prescription-only status, the reported studies suggest antibiotics are supplied without valid prescription in the studied LMICs. However, cursory knowledge, rationales and health-seeking behavior as well as social determinants of health influences the prevalence of NPA utilization across studies settings. At the same time, this represents a drawback for the health-care system, paving the way to the development of one of the major concerns of the post-antibiotic era, the AMR. Awareness of the prevalent nature of utilisation of NPA is vital in the appropriate designing of health promotion actions towards antibiotic stewardship and conservancy in LMICs.

## Recommendations

Strict implementation of restrictions on over-the-counter sales of antibiotics was reported to be effective in reducing NPA consumption in some countries [12]. There is a need to strictly use enforcement policies and guidelines on prescribing and dispensing antibiotics to address the problem in LMICs correctly. This review emphasises the importance of introducing and or intensifying health education and promotion programs on the appropriate use of antibiotics for both community and health care workers, including pharmacists. Also, accountability mechanisms and robust supervision of pharmacies should be considered.

## Data Availability

All data analysed and reported in this paper was from published literature. However, support data and summary of the data collected are provided as supplementary files.

## Abbreviations

AMR: Antimicrobial resistance
AR: Antibiotic Resistance
GNI: Gross National Income
LMICs: Low-and-middle-income countries
NPA: Non-prescribed antibiotics
PRISMA: Preferred Reporting Items for Systematic Review and Meta-Analysis
POM: Prescription Only Medicine
QUIPS: Quality in Prognosis Studies
SMA: Self-Medication of Antibiotic
UKZN: University of KwaZulu Natal
WHO: World Health Organization
